# Evaluating the psychometric properties of the iconographical falls efficacy scale (ICON-FES)

**DOI:** 10.6061/clinics/2020/e1427

**Published:** 2020-03-09

**Authors:** Ana Carolina Silva de Souza Moreira, Giovana Zarpellon Mazo, Mariluce Poerschke Vieira, Deyse Borges Machado, Fernando Luiz Cardoso, Raquel Vieira Costa de Carvalho, Roberto Moraes Cruz

**Affiliations:** I Programa de Graduacao em Ciencias do Movimento Humano, Universidade do Estado de Santa Catarina - UDESC/CEFID, Santa Catarina, SC, BR; II Universidade do Oeste de Santa Catarina - UNOESC, Chapeco, SC, BR; III Programas de Graduacao, Universidade do Estado de Santa Catarina - UDESC/CEFID, Santa Catarina, SC, BR; IV Programa de Graduacao em Ciencias do Movimento Humano e Programa de Graduacao em Educacao, Universidade do Estado de Santa Catarina - UDESC/CEFID, Santa Catarina, SC, BR; V Psicologia, Universidade Federal de Santa Catarina - UFSC, Santa Catarina, SC, BR; VI Programa de Graduacao em Psicologia, Departamento de Psicologia, Universidade Federal de Santa Catarina - UFSC, Santa Catarina, SC, BR; VII Programa de Pos-Graduacao em Psicologia, Universidade Federal de Santa Catarina, Santa Catarina, SC, BR

**Keywords:** Fear, Older Adults, Self-Efficacy, Falls, Psychometry

## Abstract

**OBJECTIVE:**

This study aims to analyze the construct and content validity of the Iconographical Falls Efficacy Scale (Icon-FES) in order to measure the fear of falling in community-dwelling older adults.

**METHODS:**

The Icon-FES was applied to 333 older adults. An exploratory factor analysis was performed to assess internal consistency. Item response theory (IRT) and confirmatory factor analysis (CFA) were used to evaluate the consistency of the questionnaire and whether it corresponded satisfactorily to the construct “concern about falling.” Concurrent validity with the Falls Efficacy Scale-International (FES-I) and convergent validity with the Senior Fitness Test (SFT) were also assessed. Receiving operator characteristic (ROC) curves were used to determine the sensitivity and specificity.

**RESULTS:**

The structural model of the 30-item and 10-item Icon-FES showed some theoretical fragility. The final model of the new short version of the Icon-FES consisted of 13 items, yielding a theoretically satisfactory structural model. Validity analyses indicated that the 13-item Icon-FES had a moderate relationship with the SFT, a strong relationship with the FES-I, and good sensitivity and specificity for a history of falls.

**CONCLUSION:**

The 13-item Icon-FES has excellent psychometric properties for measuring fear of falling in community-dwelling older adults. It can be recommended as a screening tool for fear of falling for both research and clinical purposes.

## INTRODUCTION

Fear of falling (FOF) has been defined as a continuing concern characterized by walking anxiety or excessive worry about falling, which may affect older adults by limiting their activities of daily living (ADLs) (1,2). It may also be considered a post-fall syndrome or a type of phobia (3). Over the past decades, FOF has been increasingly reported in community-dwelling older adults, with a prevalence ranging from 20 to 85% (1,4,5), being present even among those who have not experienced any type of fall (1,5). FOF should, therefore, be seen as a health problem that requires attention and preventive measures in the immediate future because the consequences of falls can have an incalculable impact on the community-dwelling older population.

FOF can be considered part of a vicious circle associated with the fall, as it can lead to decreased quality of life, increased medication use, activity restriction, further functional decline, reduced social interactions and cognitive decline (1,5). As a consequence, it further increases fall risk and is associated with an increase in the rate of premature admission to institutional care and mortality (5-7).

The accurate assessment of FOF in older adults is essential to identify those who may be at increased risk of falling, as well as to develop interventions that can effectively prevent FOF (7). However, the FOF construct is complex and involves physical, behavioral, and functional components. In this respect, Tinetti et al. (8) developed the first scale for the assessment of self-efficacy concerning falls - the Falls Efficacy Scale (FES). The FES assesses the confidence of older adults in performing a series of daily tasks without falling. In a cross-sectional study, Tinetti et al. (9) found that higher FES scores were strongly associated with reduced functional and social capacity.

However, FES has presented some limitations (10), such as the lack of complex activities that may be relevant for older people with greater functionality than the average of this population. Also, none of the FES items directly assess the impact of FOF on social life. In view of the limitations of FES, the Prevention of Falls Network Europe (ProFaNE) group developed the Falls Efficacy Scale-International (FES-I); it is composed of 16 items encompassing social activities that require different levels of postural sway in order to be performed, which are described in the literature as the main cause of fall-related concerns among older adults (11).

The FES-I has shown excellent psychometric properties in various cultural contexts (12-17). However, these studies have focused predominantly on the institutionalized older population exhibiting low physical activity levels and difficulty in performing ADLs, as well as those with musculoskeletal or neurological disorders. Consequently, their scientific validation in the general older population has been somewhat compromised.

Delbaere et al. (18) then developed the Iconographical Falls Efficacy Scale (Icon-FES) for the evaluation of older people with lower levels of concern about falling. The Icon-FES, which is geared towards high functioning older people, includes 30 activities and uses pictures to provide clear and unambiguous contexts (i.e., pictures are used as visual clues to provide more ample environmental contexts in order to facilitate the understanding of the task evaluated in relation to the FOF) (18). The Icon-FES has been cross-culturally adapted for the Brazilian population (19), but it has been validated only in terms of trustworthiness and reliability. The Icon-FES has not yet been analyzed psychometrically in terms of construct and content validity. The objectives of this study were to evaluate the psychometric properties in terms of construct and content validity of the Icon-FES for the older population living in communities.

## MATERIALS AND METHODS

### Study design

This was a prospective study of cross-cultural evaluation of the psychometric properties of the Icon-FES to measure the levels of concern about falling in community-dwelling older adults. The Ethics Committee of the local Santa Catarina State University in Brazil has approved the study (approval number: CAAE 47417715.9.0000.0118).

### Participants

A total of 333 community-dwelling older adults participated in this study. Participants were recruited from health centers where research and physical activity extension projects were taking place for the older population in the state of Santa Catarina, in city of the older adults Project in the city of Chapeco/SC; Project for the Study Group of the Third Age at the University of the State of Santa Catarina - UDESC; and Third Age groups in the Greater Florianópolis/SC metropolitan region. Eligible participants were all adults aged 60 years or older who lived in the community and were able to walk independently (with or without walking aids). Exclusion criteria were mobility, vestibular and/or cardiovascular deficits that would preclude participants from walking for 20 min with or without a walking aid, wheelchair use, presence of diseases such as stroke, Parkinson’s disease, Alzheimer’s disease, other neurological disorders, and cognitive deficits detected by the Mini-Mental State Examination (MMSE). The degree of cognitive deficit was determined according to the 23 cutoff point proposed by Manubens et al. (20).

### Measures

#### Iconographical Falls Efficacy Scale (Icon-FES)

**Figure 1:**
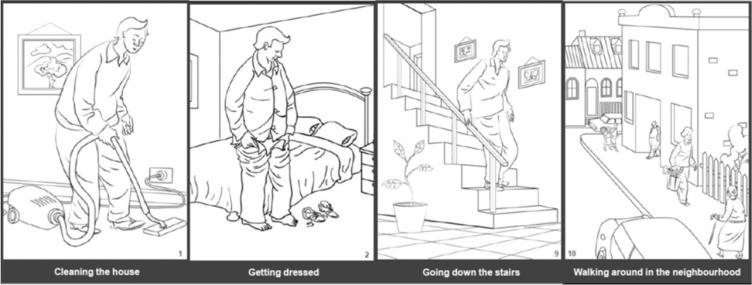
Representation of four items of the Iconographical Falls Efficacy Scale (Icon-FES).

The Icon-FES was developed as a questionnaire to be applied during interviews with older adults. It was designed to provide information on the level of concern of older people about falling relating to a series of ADLs by combining pictures of ADLs with short captions (Figure 1). The original Icon-FES contains 30 items that are scored on a 4-point scale (1=not at all concerned to 4=very concerned), for a total score ranging from 30 (corresponding to “no concern”) to 120 (corresponding to “extremely concerned about falling”) during the performance of the specific activities suggested by the questionnaire. The participants are encouraged to answer the questions based on how they habitually perform these activities. For example, if they usually use a walking aid, the response to gait-related items should show the degree of concern about falling when using such a device. If the individual does not perform a certain activity, he/she should try to imagine performing the activity in order to answer the question (18). The Icon-FES-Brazil version was used. The 30-item and 10-item Icon-FES-Brazil have shown good internal consistency (alpha and omega >0.70) and excellent intra-rater reproducibility (ICC_2,1_=0.96 and 0.93, respectively) (19).

#### Fall Efficacy Scale-International (FES-I)

The FES-I consists of 16 questions geared towards assessing the level of concern of participants about falling while performing 16 different ADLs. The FES-I items are also scored on a 4-point scale (1=not at all concerned to 4=very concerned). The total score ranges from 16 to 64, where 16 indicates “no concern” and 64 indicates “extremely concerned about falling” during the performance of the specific activities suggested by the questionnaire. As for the response categories, the word “concerned” was used to express a rational or cognitive discomfort about the possibility of falling, rather than the emotional suffering that could be reflected by terms such as “afflicted”, “anxious”, or “fearful”. Using a non-emotional term is important because some respondents are unlikely to express their emotions, as it could be seen as a sign of weakness. Also, poor self-efficacy characterized as “fear” may be a poor predictor of behavior, since “fear” carries psychiatric connotations that can imply phobia. The maximum possible score is 64. Depending on the number of responses, the older adults were classified as having a “low concern” (scoring 16 to 19), “moderate concern” (scoring 20 to 27), or “high concern” (scoring 28 to 64) about falling. This scale has demonstrated good psychometric properties (Cronbach’s alpha >0.8) (12-17).

#### Senior Fitness Test (SFT)

The SFT battery (21) consists of six motor tests that evaluate upper body strength (forearm flexion), lower body strength (30-s chair stand), upper body flexibility (back scratch), lower body flexibility (chair sit-and-reach), agility/dynamic balance (2.44-m up-and-go), and aerobic endurance (6-min walk or standing gait) according to age group. In the present study, we decided to perform the 6 minute walk test to evaluate aerobic endurance since it is the most commonly used test in Brazilian studies (22). The SFT was validated according to the three types of evidence proposed by the American Psychological Association (23), namely content, criterion, and construct validity.

### Procedures

After inclusion in the study, the older adults were individually interviewed to obtain data on sociodemographic characteristics (e.g., age, sex, marital status, level of education), health status (e.g., illness and medication), fall history, and practice of physical activity. All data were obtained from the self-report of the older adults, followed by the application of the FES-I scale through interviews. After 12 months of the first application of FES-I, the researchers were able to make personal contact with 112 older-adult participants or their caregivers to ascertain the incidence of falls during this period. Of these, 43 older adults (38%) reported having fallen at least once in the last year. The incidence of falls was defined as the unintentional contact with a bearing surface, resulting in a change in the previous position to a lower level from the initial position, without there having been a determining intrinsic factor or an unavoidable accident. There was a sample loss of 221 participants for the following reasons: participants could not be reached via telephone or were no longer taking part in the research or in the physical activity extension project to which they had been recruited (n=195); participants died (n=13); participants had moved to another state (n=10); or participants then presented mental or cognitive impairment according to their caretaker (n=3).

### Statistical analysis

A combination of categorical exploratory and confirmatory factor analyses with item response theory (IRT) methods served as the basis for the psychometric analysis of the instrument, considering the nature of the Likert scale categorical in the free software R. The “Psych package” was employed to ascertain the structural dimension of the items, using exploratory factor analysis (EFA) with principal component analysis based on a polychoric correlation matrix. Kaiser-Meyer-Olkin (KMO) and Bartlett’s sphericity tests were used to measure sampling adequacy. Cronbach’s alpha coefficient was employed to evaluate the internal consistency of the instrument for all items.

The items of each factor were later evaluated by the IRT model, in combination with the two-parameter logistic model through the “Mirt package” in the free software R. The IRT approach searches for evidence of validity based on the internal structure and consistency of the instrument. The logistic model used two parameters, *a_i_* which evaluates the discriminating power of the item and *b_i_* which evaluates the level of difficulty of the item. However, in the “Mirt package,” *b_i_* measures ease rather than difficulty. Therefore, in this package, the higher the *b_i_*, the lower the latent trait value, while the lower the *b_i_*, the higher the latent trait value.

A confirmatory factor analysis (CFA) was also performed using the software R. The “Lavaan package” was employed to ascertain whether the structural model of the questionnaire corresponded satisfactorily to the construct “concern about falling” in independent community-dwelling older adults by using the diagonally weighted least squares (WLSMV) method. However, it is specifically designed for ordinal data. Model quality was assessed for fit using Bentler’s comparative fit index (CFI), Tucker-Lewis index (TLI), root mean square error of approximation (RMSEA), and the absolute index “0χ^2^/gl” (24).

The MedCalc^®^ Statistical Software, version 17.4 (MedCalcSoftware bvba, Ostend, Belgium; http://www.medcalc.org; 2017), was used to assess the predictive validity of the total score with a sample size of 112 older adults. It was possible to verify the incidence of falls in the prospective 12-month period. The parameters used to analyze the receiving operating characteristic (ROC) curve of the test were the cutoff point, the area under the curve, the sensitivity, and the specificity. Acceptable values under the ROC curve ranged from 1 (perfect test) to 0.5, and all values below 0.5 were considered inadequate.

Statistical analyses were performed using SPSS for Windows (Version 21, IBM, Inc., Chicago, IL, USA). The data were checked for normality using the Kolmogorov-Smirnov test, which confirmed normal distribution. Discriminant validity of the Icon-FES-Brazil was assessed using Student’s t-test to control for sex, age, and history of falls. Spearman’s correlation (rho) was used for concurrent and convergent validity. A rho value of 0-0.20 indicates a very weak correlation (independence between variables), 0.21-0.29 indicates a weak correlation, 0.30-0.69 indicates a moderate correlation, 0.70-0.89 indicates a high correlation, and ≥0.90 indicates an excellent correlation (25).

## RESULTS

The study included 333 active older adults engaged in regular physical activity aged between 60 and 79 years, with a mean age of 69.6±7.1 years. Of the 333 older adults participating in the study, 90 (27%) were men and 243 (73%) were women. Fallers accounted for 25.23% (n=84) of these older adults. The mean age and level of habitual physical activity did not differ significantly between the groups of fallers and nonfallers (Table 1).

### Evaluation of the psychometric properties of the Icon-FES

#### Construct validity

Icon-FES items showed a moderate to strong correlation with each other (APPENDIX). The Icon-FES showed to be a two-dimensional instrument in the analysis of classical psychometric parameters (KMO=0.95, explained variance=61%, and Cronbach’s alpha=0.96) (Table 2).

The IRT analysis (Table 3) confirmed the two-dimensionality of Icon-FES, and all items showed good discriminating power, with a_i_ values above +1. Regarding the difficulty of the items, two items (Q19 and Q25) showed negative b_1_ values (i.e., items with lower levels of the latent trait) and three items (Q3, Q6, and Q7) had positive b1 values, indicating the presence of higher levels of the latent trait. The distance between b1 and b3 ranged from 1.14 to 2.3, indicating that the categories of the items are sufficient to obtain the necessary information about the latent trait.

CFA showed that the instrument in the unidimensional structural model of the questionnaire had a poor fit. All associations between items recommended by index modification analyses were performed (χ^2^/gl=2.89, CFI=0.884, TLI=0.869, and RMSEA=0.074). When analyzing the instrument in the two-dimensional structure, CFA did not show a good model fit. Even after performing all associations between items in order to obtain a good model fit (χ2/gl=2.77, CFI=0.883, TLI=0.880, and RMSEA=0.071), CFA indicated that the instrument presents a fragile theoretical model concerning the latent trait.

**Figure 2:**
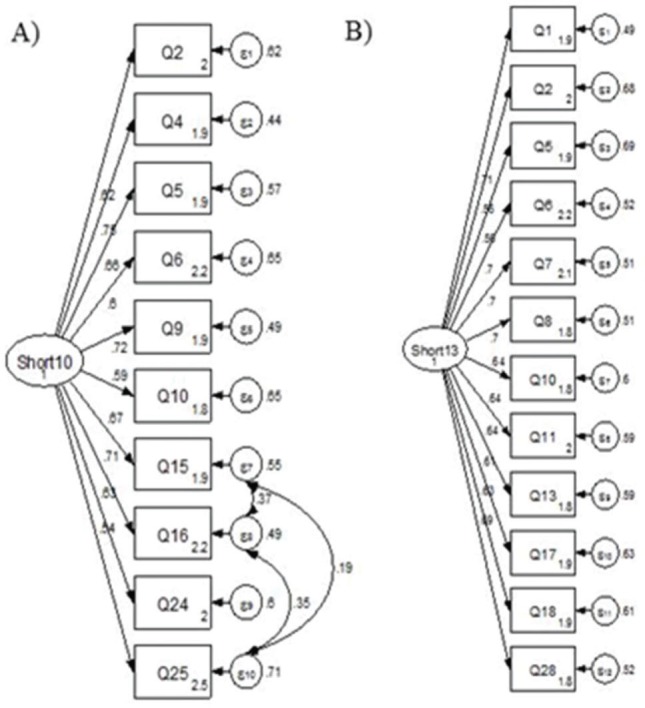
Confirmatory factorial analysis of the short Icon-FES. A) 10-item Icon-FES structural model; B) 13-item Icon-FES structural model.

When analyzing the short version of the Icon-FES with 10 items, it showed to be a unidimensional instrument in the analysis of classical psychometric parameters (KMO=0.89, explained variance=44%, and Cronbach’s alpha=0.86). The IRT analysis (Table 3) confirmed the unidimensional structural model of the 10-item Icon-FES. However, in the CFA, to obtain a good model fit, it was necessary to make associations between items Q15-Q16, Q15-Q25, and Q16-Q25 that refer to the same motor tasks involving height in order to perform the task (χ^2^/gl=7.4, CFI=0.920, TLI=0.901, and RMSEA=0.0105). Although the CFI and TLI indicate a satisfactory theoretical model, the χ2/gl and RMSEA parameters are not adequate, suggesting that the structural model of the instrument is still theoretically susceptible (Figure 2).

From the analyzed data by EFA, IRT, and CFA, the items with better factorial loads and cumulative EFA were selected. After selection by IRT of the items with good parameters of discriminating power and ability to evaluate individuals with low and high latent trait in their categories, we ultimately selected the items with better parameters of factorial load (λ) and without the association of errors (e) of the items with each other in CFA. The final model of the new short version of Icon-FES was composed of 13 items, which showed a unidimensional behavior in the EFA, with an explained variance of 62%, Cronbach’s alpha of 0.90, and KMO of 0.93. In the CFA, the model of the new version of the Icon-FES obtained a theoretically satisfactory structural model, without association between items (χ^2^/gl=3.02, CFI=0.928, TLI=0.913, and RMSEA=0.076) (Figure 2).

#### Criterion validity

The total score of the FES-I showed a significant high correlation with the total scores of the 30-item Icon-FES-Brazil (rho=0.78, *p*<0.001), of the 10-item Icon-FES (rho=0.72, *p*<0.001), and of the new short version of the Icon-FES with 13 items (rho=0.75, *p*<0.001). In addition, identical individual items from FES-I and Icon-FES-Brazil showed moderate correlations (polychoric correlation coefficient ranging from 0.35 to 0.75, *p*<0.001) (Table 4).

The new 13-item short-version of the Icon-FES was correlated with the SFT, showing a significant moderate correlation with upper body strength, lower body strength, aerobic endurance by the 6-min walk test, agility/dynamic balance, and upper body flexibility. The FES-I also correlated with the SFT, showing a significant moderate correlation with upper body strength, agility/dynamic balance, and lower body strength, but a poor correlation with upper body flexibility and aerobic endurance by the 6-min walk test (Table 5).

The area under the ROC curve in the 13-item Icon-FES was −0.74 (*p* <0.001), the cutoff point for differentiating fallers from nonfallers was 29 points, with a sensitivity of 88% (i.e., the ability to provide a positive indicator to discriminate between fallers and nonfallers) and a specificity of 72% (i.e., the ability to discriminate non-fallers among those considered fallers).

## DISCUSSION

The 30-item Icon-FES showed adequate classical psychometric parameters that confirmed its two-dimensionality, thus not being comparable to the original Icon-FES. The IRT analyses of the 30-item Icon-FES allowed for the evaluation of the scale items in order to confirm whether “concern about falling” was adequately assessed by the different tasks with different levels of difficulty. However, when using an approach based on psycho-conservatism guided by CFA, the instrument showed some theoretical fragility. The same occurred in the analysis of the 10-item Icon-FES, which also presented theoretical fragility by the CFA analysis, with a very low explained variance of 44% in the “concern about falling” construct by EFA.

The 30-item Icon-FES showed to be a two-dimensional instrument in the analysis of classical psychometric parameters, distinct from the original unidimensional scale (Icon-FES) which has a high level of association (rho=0.89). The author of the Icon-FES, however, reported in the results the two-dimensional behavior of the instrument, which checked the concern about falling while performing daily activities. The first factor prevailed tasks related to postural balance disorders, which involve greater levels of postural control. The second factor grouped the basic indoor tasks of daily living. However, when analyzing the instrument in the two-dimensional structure, CFA did not show a good model fit, even after performing all associations between items in an attempt to obtain a good model fit. The association between items suggests that the instrument has items that evaluate the same dimension of the construct. CFA showed an association between items Q2-Q4 and Q4-Q5, which are items that, despite referring to different tasks, require individuals to adopt a postural sway strategy in order to perform the task. The associations between items Q15-Q16, Q15-Q25, Q16-Q19, Q16-Q25, Q19-Q23, and Q19-Q25 refer to the same motor tasks involving height in order to perform the task. The same was observed in the associations between items Q8-Q9, which require similar motor tasks that differ only in going “up” or “down” stairs. The associations between items Q18-Q29, Q21-Q4, Q21-28, Q22-Q29, and Q27-Q29 refer to the same motor tasks involving dynamic balance and agility. These results indicate the possibility of developing a shortened version with good classical and modern psychometric parameters.

An approach of modern psychometrics through IRT and CFA analysis aims to develop not only valid but also more reliable tools that are able to measure the latent trait reliably without the need for numerous items. The analysis of the 30-item Icon-FES showed items with good discriminating power. However, CFA indicated many redundant items, which impaired the structural adjustment model of the instrument. In other words, it has redundant items without additional theoretical power. The same occurred in the 10-item Icon-FES, which presented three redundant items impairing its adjustment in the theoretical model.

Given the results of the analysis of the data from the 30-item and 10-item Icon-FES versions, it was necessary to construct a new shorter version of the Icon-FES with a satisfactory structural model and without similar items that was capable of measuring the construct “concern about falling”. Thirteen items were selected to compose the new Icon-FES version, which showed a unidimensional behavior in the EFA, with an explained variance of 62%, and a theoretically satisfactory structural model in the CFA, without association between errors (e) of items.

Based on the moderate correlation observed between the 13-item Icon-FES and functional capacity tests, it is suggested that the Icon-FES reflects the physical difficulties experienced by older adults in daily activities during which they may fall. The original Icon-FES obtained only a weak-to-moderate correlation with the functional tests of the SFT battery, suggesting that the withdrawal of items with low latent trait levels improves the representation of the phenomena by the instrument.

The 13-item Icon-FES was able to predict the risk of falls in older adults through a prospective 12-month follow-up, with a sensitivity of 88% and a specificity of 72%, and with a cutoff of 29 points. Therefore, the Icon-FES is recommended for scientific application in populations with the present sample profile. The suggested cutoff point can help establish standards for acceptable intervals of concern about falling in different populations. However, the cutoff point, as well as its predictive validity, is dependent on cultural variations and sample profiles. Caution is advised when using this cutoff point in clinical practice and clinical trials using different sampling methods or with a different cultural profile.

Additionally, this study points out that using a single clinical test to predict the risk of falls in older adults is not recommended since falling is a multifactorial phenomenon, and other risk factors need to be addressed to predict fall risk in the older population effectively. However, it is essential to know which clinical tests have discriminating power and are valid for the profile of the population to be studied. The clinical selection of instruments to appropriately evaluate fall risk should be supported by studies that have assessed such instruments for validity and reliability concerning the proposed population. Instruments in which only subcomponents have predictive validity should not be recommended for use until the instrument has been retested and validated as a whole.

The high correlation between the 13-item Icon-FES and the FES-I scales suggest that the two evaluation methods are similar, but the use of pictures in the Icon-FES may have enhanced the participants’ understanding of the items while measuring their concern about falling during the performance of the activity, thus bringing measurement closer to the actual phenomenon. Previous studies (26,27,28) suggest that providing pictures, as compared to using only words, allows individuals to better recognize and identify the situation by increasing familiarity. Therefore, using pictures in combination with words is likely to help individuals to interpret the contextual meaning correctly. In this respect, the Icon-FES may provide more details about the level of FOF by better approaching the “concern about falling” constellation in a variety of situations faced by the older population than the FES-I.

The results of this study cannot be generalized to frail older individuals with cognitive or functional impairment. Future validation studies should investigate the predictive validity of the 13-item Icon-FES and its sensitivity to detect changes in the levels of FOF over time among frail older adults, independent older adults with varying levels of functional ability, and older adults with cognitive impairment. Another aspect to consider is the fact that there may have been some memory bias influencing the data because the outcomes of interest were collected by self-report measures.

### Highlights

- The Icon-FES with 13 items can be used with confidence by researchers.
- The 13-item Icon-FES can be recommended as a screening tool for fear of falling.
- The 13-item Icon-FES can be used for both research and clinical purposes.

## CONCLUSION

The results of the present study showed that the 30-item and 10-item Icon-FES-Brazil versions did not have adequate psychometric properties for measuring FOF in community-dwelling older adults with respect to the analysis of classical parameters. The new 13-item Icon-FES version showed a theoretically satisfactory structural model in the CFA, without association between errors (e) of items.

Validity analyses indicate that the 13-item Icon-FES has a moderate relationship with the SFT, a strong relationship with the FES-I, and good sensitivity and specificity for a history of falls. Based on these analyses, we believe that the 13-item Icon-FES-Brazil can be recommended as a screening tool for FOF in older adults for both research and clinical purposes.

## AUTHOR CONTRIBUTIONS

Moreira ACSS conceived and designed the study. All authors participated in the performance of the study. Moreira ACSS and Mazo GZ participated in the recruitment and collection of older adults. Moreira ACSS, Cruz RM, and Cardoso FL were involved in the analysis and interpretation of the data. All authors contributed to revising the draft, had full access to all the data, and read and approved the final version of the manuscript. Machado DB, Mazo GZ, and Cardoso FL critically revised and completed the final draft of the manuscript.

## Figures and Tables

**Table 1:** Characteristics of participants.

	Fallers (n=84)	Nonfallers (n=249)	*p*
Women	69 (20.72%)	174 (52.25%)	0.001
Men	15 (4.5%)	75 (22.5%)	
Age	69.0±0.6	69.8±7.1	0.19
Habitual physical activity	4.4±0.4	5.1±0.2	0.21

**Table 2:** Exploratory factor analysis of the Icon-FES’ indicators.

		Icon-FES			10-item Icon-FES		13-item Icon-FES	
Item	Indicators	Factor1	Factor2	C	Factor1	C	Factor1	C
Q1	Cleaning the house	0.62	0.20	0.62			0.81	0.66
Q2	Getting dressed or undressed	0.27	0.43	0.44	0.60	0.36	0.66	0.43
Q3	Preparing simple meals	0.76	0.07	0.66				
Q4	Taking a bath	0.02	0.76	0.60	0.74	0.55		
Q5	Taking a shower	0.07	0.65	0.50	0.65	0.42	0.64	0.41
Q6	Going to the shop	0.77	0.03	0.63	0.57	0.32	0.82	0.67
Q7	Getting in or out of a chair	0.78	0.06	0.69			0.85	0.73
Q8	Going up stairs	0.22	0.64	0.68			0.78	0.60
Q9	Going down stairs	0.08	0.78	0.71	0.73	0.53		
Q10	Walking around in the neighborhood	0.58	0.19	0.55	0.58	0.34	0.75	0.56
Q11	Walking in the neighborhood in rainy weather	0.57	0.23	0.59			0.73	0.54
Q12	Walking in the neighborhood in windy weather	0.78	0.01	0.62				
Q13	Walking in the neighborhood in the dark	0.86	-0.07	0.65			0.71	0.51
Q14	Reaching for something above your head (ground)	0.59	0.22	0.61				
Q15	Reaching for something above your head (safe step)	0.04	0.77	0.64	0.72	0.52		
Q16	Reaching for something above your head (chair)	-0.12	0.98	0.80	0.78	0.60		
Q17	Reaching for something on the ground	0.21	0.53	0.50			0.74	0.55
Q18	Going to answer the telephone before it stops ringing	0.25	0.57	0.60			0.76	0.58
Q19	Walking on a slippery surface	0.090.80	0.72	0.630.63				
	(e.g., wet or icy)		-0.01					
Q20	Visiting a friend or relative	0.97	-0.14	0.76				
Q21	Walking in a place with crowds	0.44	0.38	0.60				
Q22	Walking on an uneven surface (rocks or bumpy)	0.31	0.46	0.53				
Q23	Walking down a slope	0.89	-0.04	0.74				
Q24	Going out to a social event (e.g., church, family reunion or gathering in the club)	-0.09	0.85	0.60	0.63	0.40		
Q25	Cleaning the gutter	0.51	0.17	0.43	0.59	0.35		
Q26	Stepping into the escalator	0.38	0.46	0.62				
Q27	Running to catch the bus	0.74	0.09	0.66				
Q28	Crossing the street	0.46	0.39	0.63			0.81	0.66
Q29	Crossing a busy street	0.73	0.01	0.55				
Q30	Crossing the street against the lights	0.62	0.20	0.62				
	Squared loadings	10.53	7.91		5.17		6.79	
	Proportion variance	0.35	0.26					
	Explained variance	61%			44%		62%	
	KMO	0.95			0.91		0.93	
	Cronbach’s alpha	0.96			0.88		0.90	

**Table 3:** Analysis of the items by the item response theory (IRT) model.

Items	a1	b1	b2	b3	d1	d2	d1
Q1	2.30	-2.21	-3.98	-5.64	1.76	-1.67	-3.43
Q2	1.47	-0.94	-2.92	-4.91	1.98	-1.98	-3.96
Q3	2.52	-3.98	-5.92	-7.46	1.94	-1.54	-3.47
Q4	1.91	-0.07	-2.31	-3.95	2.24	-1.64	-3.88
Q5	1.54	-0.58	-2.61	-4.14	2.03	-1.53	-3.56
Q6	2.14	-2.73	-4.82	-6.95	2.08	-2.13	-4.21
Q7	2.71	-3.27	-5.42	-6.33	2.16	-0.91	-3.06
Q8	2.41	-0.97	-3.17	-4.51	2.21	-1.34	-3.55
Q9	2.35	-0.27	-2.75	-4.41	2.48	-1.65	-4.13
Q10	2.03	-1.71	-3.22	-4.99	1.51	-1.77	-3.28
Q11	2.05	0.16	-1.83	-3.88	1.99	-2.04	-4.03
Q12	1.98	-0.28	-2.09	-3.59	1.81	-1.50	-3.30
Q13	1.94	0.17	-1.46	-3.14	1.63	-1.68	-3.31
Q14	2.12	-0.75	-2.75	-4.07	2.00	-1.32	-3.32
Q15	2.03	0.53	-1.54	-3.12	2.07	-1.58	-3.65
Q16	2.41	1.90	-0.75	-2.32	2.65	-1.57	-4.22
Q17	1.75	-1.29	-3.00	-4.54	1.71	-1.54	-3.25
Q18	2.17	-1.37	-3.38	-5.21	2.01	-1.83	-3.84
Q19	2.17	3.53	0.27	-1.50	3.25	-1.77	-5.02
Q20	2.67	-1.57	-3.87	-6.71	2.30	-2.84	-5.14
Q21	2.34	-0.94	-2.76	-4.61	1.82	-1.85	-3.68
Q22	2.36	1.93	-1.14	-3.47	3.07	-2.33	-5.40
Q23	1.95	0.16	-2.16	-3.89	2.32	-1.73	-4.05
Q24	2.66	-2.25	-4.29	-6.04	2.04	-1.75	-3.79
Q25	1.73	2.00	0.87	-0.24	1.13	-1.10	-2.23
Q26	1.47	-0.55	-2.32	-3.16	1.77	-0.84	-2.61
Q27	2.25	0.18	-1.55	-3.08	1.73	-1.53	-3.26
Q28	1.74	-0.77	-2.52	-3.79	1.74	-1.27	-3.01
Q29	1.98	1.03	-0.78	-2.25	1.82	-1.47	-3.29
Q30	1.33	0.89	-0.63	-1.57	1.52	-0.94	-2.46

Legend: a1: level of discrimination; b: level of difficulty; b1: indicates the inflection point of the curve between the first and the second category. b2: indicates the inflection point of the curve between the second and third categories. b3: indicates the inflection point of the curve between the third and last category; d1: distance between the first and second inflection points; d2: distance between the second and third inflection points; d3: distance between the first and third inflection points.

**Table 4:** Correlation between similar items on the Icon-FES and FES-I scales.

FES-I		Icon-FES-Brazil	r
Cleaning the house (e.g., sweep, vacuum, dust)	Q4	Cleaning the house (e.g., mop, vacuum or dusting)	0.40
Getting dressed or undressed	Q1	Getting dressed or undressed	0.53
Preparing simple meals	Q5	Preparing simple meals	0.53
Taking a bath or shower	Q2	Taking a bath	0.50
	Q3	Taking a shower	0.72
Going to the shop	Q15	Going to the shop	0.53
Getting in or out of a chair	Q11	Getting in or out of a chair	0.56
Going up or downstairs	Q12	Going upstairs	0.53
	Q13	Going downstairs	0.60
	Q16	Step into the escalator	0.40
Walking around in the neighborhood	Q17	Walking around in the neighborhood	0.44
Reaching for something above your head or on the ground	Q6	Reaching for something on the ground	0.37
	Q7	Reaching for something above your head (chair)	0.44
	Q8	Reaching for something above your head (ground)	0.57
	Q9	Reaching for something above your head (safe step)	0.35
Going to answer the telephone before it stops ringing	Q14	Going to answer the telephone before it stops ringing	0.57
Walking on a slippery surface (e.g., wet or icy)	Q19	Walking on a slippery surface (e.g., wet or icy)	0.45
Visiting a friend or relative	Q26	Visiting a friend or relative	0.54
Walking in a place with crowds	Q28	Walking in a place with crowds	0.75
Walking on an uneven surface (e.g., rocky round, poorly maintained pavement)	Q20	Walking on an uneven surface (e.g., rocky ground, poorly maintained pavement)	0.71
Walking up or down a slope	Q18	Walking down a slope	0.65
Going out to a social event (e.g., religious service, family gathering, or club meeting)	Q27	Going out to a social event (e.g., religious service, family gathering, or club meeting)	0.69

**Table 5:** Correlation of the Senior Fitness Test (SFT) with the 13-item Icon-FES and FES-I scales.

SFT	13-item Icon-FES	FES-I
Upper body strength	−0.39**	−0.32**
Lower body strength	−0.42**	0.34**
6-min walk test	−0.32*	0.21*
Agility/dynamic balance	0.45**	0.34**
Upper body flexibility	−0.32*	0.21*

Legend:

* significance level: *p*<0.05;

** significance level: *p*<0.001.

## APPENDIX

**Table:** Correlation between similar items on the Icon-FES and FES-I Scales.

FES-I		Icon-FES-br	r
Cleaning the house (e.g. sweep, vacuum, dust)	Q1	Cleaning the house (eg, mop, vacuum or dusting)	0.40
Getting dressed or undressed	Q2	Getting dressed or undressed	0.53
Preparing simple meals	Q3	Preparing simple meals	0.53
Taking a bath or shower	Q4	Taking a bath	0.50
	Q5	Taking a shower	0.72
Going to the shop	Q6	Going to the shop	0.53
Getting in or out of a chair	Q7	Getting in or out of a chair	0.56
Going up or down stairs	Q8	Going up stairs	0.53
	Q9	Going down stairs	0.60
	Q26	Step into the escalator	0.40
Walking around in the neighborhood	Q10	Walking around in the neighborhood	0.44
Reaching for something above your head or on the ground	Q17	Reaching for something on the ground	0.37
	Q16	Reaching for something above your head (chair)	0.44
	Q15	Reaching for something above your head (safe step)	0.35
	Q14	Reaching for something above your head (ground)	0.57
Going to answer the telephone before it stops ringing	Q18	Going to answer the telephone before it stops ringing	0.57
Walking on a slippery surface (e.g. wet or icy)	Q19	Walking on a slippery surface (e.g. wet or icy)	0.45
Visiting a friend or relative	Q16	Visiting a friend or relative	0.54
Walking in a place with crowds	Q29	Walking in a place with crowds	0.75
Walking on an uneven surface (e.g. rocky round, poorly maintained pavement)	Q26	Walking on an uneven surface (e.g. rocky ground, pooly maintained pavement)	0.71
Walking up or down a slope	Q23	Walking down a slope	0.65
Going out to a social event (e.g. religious sevice, family gathering, or club meeting)	Q24	Going out to a social event (e.g. religious service, family gathering, or club meeting)	0.69

## References

[1] Dierking L, Markides K, Al Snih S, Kristen Peek M (2016). Fear of Falling in Older Mexican Americans: A Longitudinal Study of Incidence and Predictive Factors. J Am Geriatr Soc.

[2] Halvarsson A, Franzén E, Ståhle A (2013). Assessing the relative and absolute reliability of the Falls Efficacy Scale-International questionnaire in elderly individuals with increased fall risk and the questionnaire’s convergent validity in elderly women with osteoporosis. Osteoporos Int.

[3] Lopes KT, Costa DF, Santos LF, Castro DP, Bastone AC (2009). Prevalence of fear of falling among a population of older adults and its correlation with mobility, dynamic balance, risk and history of falls. Rev Bras Fisioter.

[4] Antes DL, d’Orsi E, Benedetti TR (2013). Circumstances and consequences of falls among the older adults in Florianopolis. Epi Floripa Aging 2009. Rev Bras Epidemiol.

[5] Uemura K, Shimada H, Makizako H, Doi T, Tsutsumimoto K, Lee S (2015). Effects of Mild Cognitive Impairment on the Development of Fear of Falling in Older Adults: A Prospective Cohort Study. J Am Med Dir Assoc.

[6] Deshpande N, Metter EJ, Lauretani F, Bandinelli S, Guralnik J, Ferrucci L (2008). Activity restriction induced by fear of falling and objective and subjective measures of physical function: a prospective cohort study. J Am Geriatr Soc.

[7] Visschedijk J, Van Balen R, Hertogh C, Achterberg W (2013). Fear of falling in patients with hip fractures: prevalence and related psychological factors. J Am Med Dir Assoc.

[8] Tinetti ME, Richman D, Powell L (1990). Falls efficacy as a measure of fear of falling. J Gerontol.

[9] Tinetti ME, Mendes de Leon CF, Doucette JT, Baker DI (1994). Fear of falling and fall-related efficacy in relationship to functioning among community-living elders. J Gerontol.

[10] Yardley L, Beyer N, Hauer K, Kempen G, Piot-Ziegler C, Todd C (2005). Development and initial validation of the Falls Efficacy Scale-International (FES-I). Age Ageing.

[11] Delbaere K, Close JC, Mikolaizak AS, Sachdev PS, Brodaty H, Lord SR (2010). The Falls Efficacy Scale International (FES-I). A comprehensive longitudinal validation study. Age Ageing.

[12] Jorstad EC, Hauer K, Becker C, Lamb SE, ProFaNE (2005). Measuring the psychological outcomes of falling: a systematic review. J Am Geriatr Soc.

[13] Kwan MM, Tsang WW, Close JC, Lord SR (2013). Development and validation of a Chinese version of the Falls Efficacy Scale International. Arch Gerontol Geriatr.

[14] van Vliet R, Hoang P, Lord S, Gandevia S, Delbaere K (2013). Falls efficacy scale-international: a cross-sectional validation in people with multiple sclerosis. Arch Phys Med Rehabil.

[15] Jonasson SB, Nilsson MH, Lexell J (2014). Psychometric properties of four fear of falling rating scales in people with Parkinson’s disease. BMC Geriatr.

[16] Baharlouei H, Salavati M, Akhbari B, Mosallanezhad Z, Mazaheri M, Negahban H (2013). Cross-cultural validation of the Falls Efficacy Scale International (FES-I) using self-report and interview-based questionnaires among Persian-speaking elderly adults. Arch Gerontol Geriatr.

[17] Halaweh H, Svantesson U, Rosberg S, Willen C (2016). Cross-Cultural Adaptation, Validity and Reliability of the Arabic Version of the Falls Efficacy Scale-International (FES-I). Med Princ Pract.

[18] Delbaere K, Smith ST, Lord SR (2011). Development and initial validation of the Iconographical Falls Efficacy Scale. J Gerontol A Biol Sci Med Sci.

[19] Franco MR, Pinto RZ, Delbaere K, Eto BY, Faria MS, Aoyagi GA (2018). Cross-cultural adaptation and measurement properties testing of the Iconographical Falls Efficacy Scale (Icon-FES). Braz J Phys Ther.

[20] Manubens JM, Martínez-Lage P, Martínex-Lage JM, Larumbe R, Muruzábal J, Martínez-Gonzáles MA (1998). [Variation of Mini-Mental-State examination scores due to age and educational level. Normalized data in the population over 70 years of age in Pamplona]. Neurologia.

[21] Rikli RE, Jones CJ (1999). Development and validation of a functional fitness test for community-residing older adults. J Aging Phys Activity.

[22] Streit IA, Mazo GZ, Virtuoso JF, Menezes EC, Goncalves E (2011). Aptidão física e ocorrência de quedas em idosos praticantes de exercícios físicos. Rev Bras Ativ Fis Saude.

[23] American Psychological Association Standards for Educational and Psychological Testing (1985).

[24] Maroco J (2010). Análise de equações estruturais.

[25] Finney DJ (1980). Statistics for biologists London Chapman and Hall.

[26] Ally BA, McKeever JD, Waring JD, Budson AE (2009). Preserved frontal memorial processing for pictures in patients with mild cognitive impairment. Neuropsychologia.

[27] Westerberg CE, Paller KA, Weintraub S, Mesulam MM, Holdstock JS, Mayes AR (2006). When memory does not fail: familiarity-based recognition in mild cognitive impairment and Alzheimer’s disease. Neuropsychology.

[28] Hoang OT, Jullamate P, Piphatvanitcha N, Rosenberg E (2017). Factors related to fear of falling among community-dwelling older adults. J Clin Nurs.

